# Autobiographical narratives in major depression: changes in memory specificity during outpatient psychodynamic psychotherapy

**DOI:** 10.1186/s12888-025-07666-7

**Published:** 2025-12-06

**Authors:** Magdalena Lutz, Jonathan R. Nowak, Ivo Dönnhoff, Hans-Christoph Friederich, Valentin Terhoeven, Christoph Nikendei

**Affiliations:** 1 Department of General Internal Medicine and Psychosomatics, Center for Psychosocial Medicine, Heidelberg University Hospital, Thibautstraße 4, 69115 Heidelberg, Germany; 2 DZPG (German Centre for Mental Health – Partner Site Heidelberg/ Mannheim/ Ulm), Heidelberg, Germany

**Keywords:** Overgeneral autobiographical memory, Autobiographical memory test, Psychotherapy, Depression, Adverse childhood experiences

## Abstract

**Background:**

Previous studies have shown that patients with depression recall fewer specific autobiographical memories, a phenomenon known as overgeneral autobiographical memory (OGM). OGM refers to the retrieval of categorical memories (repeated events) and extended memories (events lasting more than 24 h), rather than specific, single-event recollections. This pattern has been linked to dysfunctional emotion regulation and childhood trauma. While most research has used the Autobiographical Memory Test (AMT) to assess OGM, such standardized cue-word paradigms are limited in capturing how autobiographical memories unfold in real-life psychotherapeutic settings. This study introduces a novel methodology to assess autobiographical memories as they naturally emerge during videotaped psychodynamic psychotherapy sessions.

**Methods:**

We analyzed videotapes of the first and 40th psychodynamic psychotherapy sessions of 55 patients with major depression. Therapist questions served as prompts for autobiographical narratives, which were rated for specificity and overgeneralization (extended, categorical). We also examined the role of adverse childhood experiences (ACEs) in changes to memory specificity during therapy.

**Results:**

Linear regression analysis showed a significant time × memory-type × ACE interaction. No significant changes were observed for categorical or extended memories. In contrast, for specific memories, higher ACE scores predicted fewer specific memories at baseline but greater increases in specificity from session 1 to 40.

**Conclusions:**

This study demonstrates the feasibility of assessing autobiographical memory specificity directly within psychodynamic psychotherapy sessions. The proposed methodology provides an ecologically valid alternative to traditional lab-based assessments of autobiographical memory and can be applied in the naturalistic context of real-life therapeutic interactions. Our findings suggest a potential improvement in autobiographical memory specificity during psychodynamic psychotherapy – particularly among individuals with higher ACE scores – that may be reliably assessed using this novel approach.

**Clinical trial number:**

Not applicable.

**Supplementary Information:**

The online version contains supplementary material available at 10.1186/s12888-025-07666-7.

## Introduction

Depression is one of the most common mental disorders and, according to recent epidemiological evidence, the second leading cause of the global burden of disease in terms of years lived with disability [1]. Major depression is characterized by a variety of emotional and somatic symptoms including depressed mood and loss of interest in activities [2]. Depression has been shown to cause significant distress, resulting in an increased risk of suicide that is more than 8% higher than in the general population [3]. Given the substantial socioeconomic impact associated with significant direct and indirect costs [4], a comprehensive understanding of the mechanisms underlying depression is essential for the development of more effective prevention and treatment strategies.

In recent years, there has been considerable interest in autobiographical memory (AM) in patients diagnosed with depression, as well as in a phenomenon termed overgeneral autobiographical memory (OGM) [5]. The concept of OGM was first introduced in suicide attempters [6] and subsequently validated in depressed individuals [7]. In the aforementioned studies and numerous subsequent investigations, patients with depression reported a higher incidence of OGM than healthy individuals [8, 9]. In AM research, *specific* memories are defined as events that occurred on a particular day (e.g., “I climbed a mountain with my uncle last Sunday”) [10]. In contrast, OGM includes *extended* (events that last longer than a day, e.g., “Last summer I spent a week in the mountains”) and *categorical* memories (events that occur repeatedly, e.g., “Every year I go hiking with my uncle in the summer”). They are distinguished from *semantic associate*s, which reflect knowledge about the self (e.g., “I like hiking”) [10]. Numerous studies have shown that patients with depression tend to retrieve fewer specific memories and more general memories [5]. This pattern can be theoretically explained by mechanisms such as capture and rumination, functional avoidance, and reduced executive capacity, as outlined in the CaR-FA-X model [8]. However, OGM was also found in other psychiatric and psychosomatic disorders [11] such as schizophrenia spectrum disorder [12], posttraumatic stress disorder [13, 14], borderline personality disorder [15], anxiety disorders [16], and anorexia nervosa [17–19]. Although most pronounced in clinical populations, similar OGM patterns have been reported in non-clinical individuals exposed to stress or cognitive load, indicating a broader relevance beyond psychopathology [20].

OGM has been conceptualized as a dysfunctional emotion regulation strategy [5]. One factor that appears to play a role in the pathogenesis of OGM is the experience of traumatic events, particularly during childhood [21, 22]. Whether OGM precedes depression or depression leads to OGM is a widely studied topic, and several studies have explored whether OGM can be altered or whether it persists following recovery from depression. A number of studies have suggested that OGM has the potential to predict the course of depression and that memory specificity can increase and OGM decrease with symptom improvement and therapeutic intervention [23–25]. However, recent meta-analytic evidence indicates that reduced AM specificity tends to persist even after remission from depression, potentially increasing the risk of relapse [5, 26].

In recent years, a number of therapeutic training programs have been developed with a specific focus on reducing OGM. Among these, memory flexibility training (MemFlex) [27] and memory specificity training (MEST) [28] have shown the most promise, although their effects have only been observed in the short term [29]. However, improvement in OGM does not necessarily correlate with improvement in depressive symptoms or relapse prevention [27]. The efficacy of common therapeutic approaches for depression, including cognitive behavioral psychotherapy (CBT) and psychotropic medications, in reducing OGM is a topic of ongoing debate in the literature [30–32]. Despite extensive research on AM impairments in depression, findings suggest that these impairments may persist even after symptomatic remission [26], and the potential role of psychotherapy in addressing them remains insufficiently understood.

One psychotherapeutic approach that places particular emphasis on autobiographical themes is psychodynamic psychotherapy. Rooted in psychoanalytic theory, it focuses on intrapsychic and interpersonal dynamics, emotional experience, defense mechanisms, and the influence of early life experiences on current behavior and psychological difficulties [33, 34]. Empirical evidence supports the efficacy of psychodynamic psychotherapy in both short- and long-term treatments across various mental disorders, including major depressive disorder [35]. Although biographical themes are central to psychodynamic work, no previous studies have examined whether AM specificity changes over the course of psychodynamic psychotherapy.

The most commonly used instrument for assessing OGM is the Autobiographical Memory Test (AMT) [6]. In this assessment, participants are presented with cue words and are then asked to recall a specific AM within a time frame of 30 to 60 s. Despite the existence of several versions of the AMT [36, 37], the predominant use of a single method in this area of research has been criticized [5, 38]. Previous studies have primarily examined changes in OGM as a result of psychotherapy using the AMT before and after therapy [30, 39]. So far, only two studies [40, 41] have used psychotherapy sessions as a means to study AM. They followed a thematic approach in brief emotion-focused and client-centered treatments for depression, using the narrative process coding system [42] to identify discrete emotional episodes [43] and to assess the AMs reported here. While these two studies [40, 41] examined AMs elicited during moments of heightened affective arousal, they do not capture how AM is used across the full breadth of therapeutic discourse. By focusing exclusively on emotionally charged moments, they may overlook autobiographical remembering in more neutral contexts, which could yield important insights into everyday therapeutic processes.

Although the AMT has been widely used to assess memory specificity, it offers a relatively narrow view of AM. Its use of isolated cue words and time-limited responses may restrict the depth, context, and emotional engagement typically involved in naturally occurring memory recall [38]. In contrast, psychotherapy provides a socially and emotionally rich environment in which AMs are recalled in response to open-ended, relationally embedded prompts and in the presence of a responsive listener [34, 44]. Therapist-related prompts in this setting are grounded in the patient’s discourse and therapeutic goals, potentially allowing for more elaborated, personally meaningful, and contextually grounded memories to emerge. Studying memory in socially embedded contexts may offer valuable insights into the role of interpersonal processes in AM – an aspect often overlooked by standardized experimental tasks.

The aim of the present study was to develop a method for analyzing OGM in the naturalistic setting of psychotherapy sessions. To this end, we assessed OGM within videotaped psychodynamic psychotherapy sessions, which provides a more ecologically valid perspective and complements findings from traditional memory assessments like the AMT. In contrast to the aforementioned studies [40, 41], narratives of all types of emotional valence and arousal were included, and therapist questions, arising naturally from routine psychotherapy dialogue rather than being standardized, were used as prompts to identify potential autobiographical narratives.

In addition, we sought to determine whether adverse childhood experiences (ACEs) could predict changes in the specificity of the reported AMs over the course of treatment as a stable baseline predictor. ACEs, including emotional, physical, and sexual abuse or neglect [45], have been consistently associated with reduced AM specificity across the lifespan, including in children, adolescents, and adults [21, 46, 47]. Theoretical models suggest that trauma-related mechanisms – such as avoidance, impaired encoding, and fragmented memory processing – may underlie AM overgeneralization [48]. These processes can hinder the retrieval of specific memories and impact how memory specificity changes over time, particularly in the context of psychotherapy. Understanding the role of ACEs is therefore critical when examining trajectories of AM specificity in clinical populations.

We hypothesized that such a study design would be feasible and that AM specificity would increase while OGM would decrease over the course of psychotherapy. Furthermore, we hypothesized that ACEs would influence changes in AM specificity over the course of treatment. However, due to the absence of prior research on this association in the real-life context of psychotherapy sessions, we did not specify a directional hypothesis.

## Methods

### Part 1: Methodological approach and its development

#### Study aim

The purpose of this study was to develop a methodological approach for assessing autobiographical memory (AM) from videotaped outpatient psychodynamic psychotherapy sessions. Inspired by the Autobiographical Memory Test (AMT) [6], which uses cue words to prompt patient responses, we used therapist questions as anchors to elicit autobiographical narratives. Similar to the AMT, in which the time to elicit a memory is limited [6], we introduced a time criterion of 30 s as a cutoff for the inclusion of patient narratives. The entire sessions were analyzed and, for each therapist question, a 30-second window following the question was used to identify AM retrieval. This time span seemed reasonable, since in most of the patient turns AMs appeared within the first 30 s.

In this study, the term *autobiographical memory* refers to discrete memory content units that were coded for specificity (i.e., specific, extended, categorical). The term *autobiographical narrative* is used when referring to the broader verbal context in which such memories were embedded within the therapeutic conversation. For clarity, we use *memory* throughout the manuscript when describing the coded units used in quantitative analyses.

### Therapist questions used as prompts to elicit autobiographical narratives

Analogous to existing classification systems from conversation analysis [49–53] and the Psychodynamic Interventions List (PIL) [54], we developed a classification of therapist questions based on the video material of the recorded therapy sessions. The codes for the question types were developed following a multi-step approach over an extended period. In weekly meetings of our research group, preliminary coding drafts were critically reviewed and continuously refined, ultimately leading to the final coding scheme. Table 1 shows the developed classification with a brief definition and short examples of the question types.

**Table 1:** Classification of therapist questions used as cues for autobiographical memories (AMs)

Question type	Definition	Example
Open-ended question	Questions that allow the patient to choose the topic. Often the first question in the therapy session, opening the conversation.	“So, how are you doing?“
Focus on feelings	Questions that address general or situational feelings. Past and present emotions may be of interest. The words “feeling” or “emotion” alone are not sufficient if they are not addressed.	“And how do you feel about being promoted to management?”
Reflection on current AM	This question type aims to explore the current AM in more detail to get the patient to reflect on it. The patient is most likely already thinking about an AM based on the previous conversation.	“What is new about it?“
Addressing new topic	Question type that elicits an AM when the patient is unlikely to be thinking of an AM.	“So, are there any aspects of yourself that you like?”
Asking for specification	The therapist asks for more specific information or examples about a previously superficially discussed topic.	“What do you mean by ‘little things at home’?“
Asking for generalization	The therapist asks for a general statement or a similar experience derived from a specific situation. In terms of memory, a specific AM is linked to another by an associative criterion that can be of any specificity.	“You’ve done this before, haven’t you? Recently, a few months ago?“

### Rating of patients’ responses

MAXQDA 2020 [55] was used to analyze the autobiographical narratives. The coding was performed manually, without the use of algorithms, in two steps: First, patient utterances that followed a therapist question were identified. In a second step, these were rated for memory specificity using the audio tracks of the videos. The rating of AM specificity was performed by distinguishing between “no AM”, “semantic associate” (knowledge about the self), “categorical AM” (memories of events that occur repeatedly), “extended AM” (memories of events that last longer than one day) and “specific AM” (memories of events that occurred on a particular day).

For a patient utterance to qualify as AM, it had to describe a personally experienced past event with clear mental involvement. Memories did not require exact temporal markers but had to refer to a specific day (specific AM), a repeated pattern (categorical AM), or a prolonged episode (extended AM). Dreams were also counted as AMs. Semantic statements were included only when they clearly referred to past personal experiences (semantic associates). Utterances referring to events that occurred after entering the therapy room, memories of other people’s experiences, or abstract reflections on current mental states were excluded. Repeated mentions of previously rated memories were not rated again unless new, relevant information altered their specificity level. In cases of multiple memories within a 30-second segment, the memory with the highest specificity or greatest narrative relevance was selected. The detailed guideline that was used for the AM evaluation procedure is provided as Additional file (see Additional file 1). The primary coding was carried out by one of the authors (ML). Rating was trained on 10 videos and difficult cases were discussed within our research group before applying the scheme on all videos.

### Interrater reliability

To assess interrater reliability (*IRR*), a second independent rating was performed by a trained rater (JN) on a subset of 30 videotaped sessions comprising a total of 637 memory sequences. Gwet’s Agreement Coefficient (*AC*) was used to quantify interrater agreement, implemented via the irrCAC package in R [56]. Ratings were based on a five-point scale ranging from 1 (no memory) to 5 (specific memory).

First, an unweighted version of Gwet’s *AC* was computed across all five rating levels, yielding *AC1* = 0.57 (95% *CI* [0.522, 0.615], *SE* = 0.0235, *p* < .001), indicating moderate agreement according to Altman’s (1990) classification [57] (0.41–0.60 = moderate).

Second, a custom-weighted version of Gwet’s *AC* was calculated to account for the degree of disagreement between adjacent categories: disagreements between directly adjacent categories (e.g., 2 = semantic vs. 3 = categorical) were weighted with 0.25, while disagreements between more distant but still neighboring categories (e.g., 1 = no memory vs. 2 = semantic; 3 = categorical vs. 4 = extended) were weighted with 0.5. All other disagreements received a weight of 0. This yielded a coefficient of *AC* = 0.62 (95% *CI* [0.579, 0.665], *SE* = 0.0221, *p* < .001), reflecting good agreement (Altman, 1990 [57]: 0.61–0.80 = good).

Third, to assess agreement on a simplified categorization of memory specificity, a three-level coding scheme was applied: (0) no memory or semantic associate, (1) overgeneral memories (categorical, extended), and (2) specific memories. Based on this coding, an unweighted version of Gwet’s *AC* yielded *AC1* = 0.69 (95% *CI* [0.646, 0.739], *SE* = 0.0238, *p* < .001), also indicating good agreement.

Finally, for a binary classification (non-specific memory responses [ratings 1–4] vs. specific memories [rating 5]), agreement was *AC1* = 0.84 (95% *CI* [0.795, 0.876], *SE* = 0.0207, *p* < .001), indicating very good agreement (Altman, 1990 [57]: >0.80 = very good).

#### Part 2: Study procedure and analysis strategy

### Study design

The study was designed as a retrospective analysis of videotaped psychodynamic psychotherapy sessions conducted at the Heidelberg Institute for Psychotherapy (HIP), Heidelberg University Hospital, Germany, a postgraduate training institute for psychologists and physicians specializing in psychotherapy that places a strong emphasis on psychodynamic psychotherapy [58]. The selected therapy sessions took place between September 2014 and January 2020. A total of 36 therapists were involved in the study. The mean interval between sessions T1 and T40 was 382.1 days (range: 273–545 days). While therapy was typically delivered on a weekly basis, longer treatment durations resulted from session cancellations due to illness or vacation. Sessions lasted 50 min and were conducted by therapists in advanced clinical training. All trainees received continuous supervision by licensed and highly experienced psychotherapists. Psychodynamic psychotherapy was chosen as the treatment framework because it emphasizes the exploration of autobiographical experiences and past interpersonal patterns. This therapeutic focus provides a naturalistic context in which AMs are likely to emerge spontaneously.

### Inclusion criteria

All patients consented to the use of their data for study purposes before the start of treatment. Inclusion criteria for our study were a diagnosis of major depressive disorder according to DSM-IV [59] and the availability of the first (T1) and fortieth (T40) therapy sessions as video recordings (alternatively the 39th or 41st session). Of the *n* = 62 patients who met the above criteria, data sets of *n* = 55 patients were selected for further analysis after excluding video recordings of insufficient quality (*n* = 6) and one set of recordings (*n* = 1) because the respective therapist is also an author of this paper. Diagnoses were established prior to the start of therapy using the Structured Clinical Interview for DSM-IV [60, 61]. The study was approved by the Ethics Committee of the Medical Faculty of the Heidelberg University (No. S-195/2014).

### Sample characteristics and procedure

Of the *n* = 55 patients, 36 (65.5%) were female and 19 (34.5%) were male. The mean age of the participants was *M* = 34.98 years (*SD* = 12.37), ranging from 18 to 61 years. A range of comorbidities were present among the patients, including anxiety disorders in 35 patients (63.6%), somatoform disorders in 7 patients (12.7%), eating disorders in 5 patients (9.1%), pain disorders in 4 patients (7.3%), substance use disorders in 3 patients (5.5%), and obsessive-compulsive disorders in 3 patients (5.5%). Of the at least 60 therapy sessions conducted per patient, the first (T1) and the fortieth (T40) were selected for this study to ensure adequate temporal spacing for the development and evaluation of therapeutic effects. All patients had completed 5 probationary sessions prior to T1, so that the content of the sessions examined consisted of therapeutically relevant and not merely organizational topics. T40 was purposefully selected as a later, but not final, therapy session to avoid focusing solely on the conclusion of therapy.

### Questionnaires

All patients completed a battery of questionnaires at baseline and at the end of treatment as part of the standard HIP assessment procedure: The Questionnaire for the Assessment of Adverse and Protective Childhood Experiences (APC) [62], Beck’s Depression Inventory (BDI) [63], the revised version of the Experiences in Close Relationships Questionnaire (ECR-RD) [64], the Inventory of Interpersonal Problems (IIP-32) [65], the Outcome Questionnaire (OQ-45) [66], the Patient Health Questionnaire (PHQ-D) [67], and the Short Form Health Survey (SF-36) [68].

### Statistical measures

Statistical analyses were performed with IBM SPSS Statistics version 25 [69] and R version 4.4.2 [70]. Paired *t*-tests were used to compare questionnaire scores from T1 and T40. To analyze the distribution of AMs within therapy sessions, sequences in which no AM was found were excluded. The number of specific AMs, extended AMs, categorical AMs, and semantic associates in T1 was added to calculate the number of all memory responses in T1. Similar calculations were performed for T40. The percentage of different categories of AMs within the therapy session was calculated by dividing the number of each category by the number of all memory responses (specific + extended + categorical + semantic) in the session.

To examine changes in AM types over time as a function of adverse childhood experiences (ACEs), we conducted a linear regression including time (T1 vs. T40), memory type (specific, categorical, extended), and ACE scores as fixed effects, along with all interaction terms. To decompose significant interactions, we estimated marginal means for each time × memory-type combination and conducted pairwise contrasts. In addition, difference-in-differences contrasts were computed to compare changes in each memory type across time (e.g., change in specific memories vs. change in extended or categorical memories). For moderation analyses, simple slopes of baseline ACE scores were estimated within each time × memory-type combination. Pairwise differences between slopes were tested using Tukey-adjusted contrasts. To assess whether symptom improvement was associated with variation in memory specificity, we extended the initial model by including change in depressive symptoms (BDI post-treatment – BDI pre-treatment) as an additional covariate. To explore whether changes in ACE scores were associated with changes in AM specificity, we ran an additional model replacing baseline ACE scores with change scores (ACE post-treatment – ACE pre-treatment). To control for potential variation in narrative output across sessions, the total number of analyzed sequences (specific AMs + extended AMs + categorical AMs + semantic associates + no AMs) per session was included as a covariate in an additional control analysis.

## Results

### Frequencies of different types of autobiographical memories

**Fig. 1:**
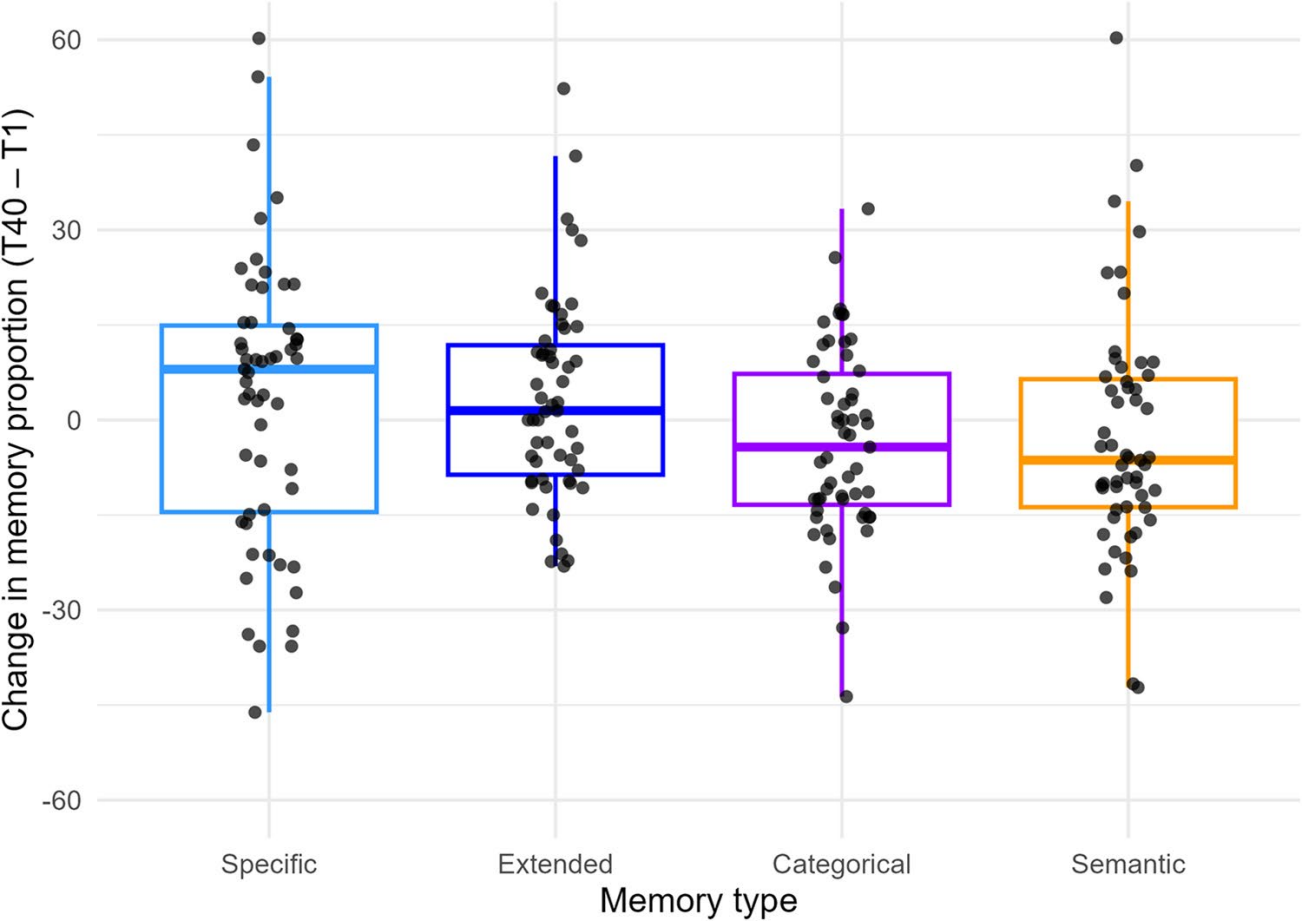
Individual change in memory type proportions between T1 and T40. Boxplots depict individual change scores (T40 minus T1) in memory proportions for each memory type: specific, extended, categorical, and semantic. Each dot represents one participant. Positive values indicate an increase, and negative values a decrease in the respective memory category across therapy sessions

At T1, a mean of 28.3 (*SD* = 13.0) sequences per therapy session were analyzed, with 66.1% (*SD* = 15.0) of the sequences showing a memory response (specific, extended, categorical, semantic). At T40, a mean of 22.1 (*SD* = 10.8) sequences were analyzed per therapy session, with 60.5% (*SD* = 18.0) of the sequences showing memory responses. There were significantly more narrative sequences analyzed at T1 than at T40 (*t* 54 = 5.04; *p* < .001). In addition, the proportion of sequences representing memory responses was higher at T1 than at T40 (*t* 54 = 6.25; *p* < .001). Figure 1 depicts the individual change in memory responses between T1 and T40.

### Questionnaires

As shown in Table 2, all questionnaires except the ECR-RD showed a significant change in patients’ symptoms. Scores for depression (BDI, PHQ), total symptom burden (OQ-45), interpersonal problems (IIP-32), and emotional well-being (SF-36) decreased significantly, suggesting that the treatment was effective overall. In addition, patients reported significantly more adverse and fewer protective childhood experiences (APC) post-treatment compared to pre-treatment.

**Table 2:** Pre- and post-treatment changes in questionnaire scores

	Pre-treatment		Post-treatment		t(54)	*p* ^a^	Cohen’s d
	M	SD	M	SD			
BDI	20.6	9.6	14.0	10.6	4.43	< 0.001	0.60
PHQ depression	11.9	5.3	7.3	5.3	5.45	< 0.001	0.52
OQ45 total	76.7	18.7	62.6	28.7	4.09	< 0.001	0.56
APC adverse	0.9	0.6	1.0	0.7	3.68	< 0.001	0.50
APC protective	2.4	0.9	2.0	1.0	5.76	< 0.001	0.78
ECR anxiety	3.7	1.3	3.7	1.5	0.16	0.436	0.02
ECR avoidance	2.6	1.0	2.8	1.0	1.37	0.088	0.19
IIP32 total	1.6	0.4	1.4	0.6	2.56	0.007	0.35
SF36 physical functioning	83.5	21.1	86.2	17.9	1.51	0.069	0.21
SF36 emotional wellbeing	41.6	14.4	58.9	20.8	6.28	< 0.001	0.85

^a^ We used one-tailed tests because we expected patients’ symptoms to improve during therapy

### Change of autobiographical memories in relation to adverse childhood experiences (ACEs)

**Fig. 2:**
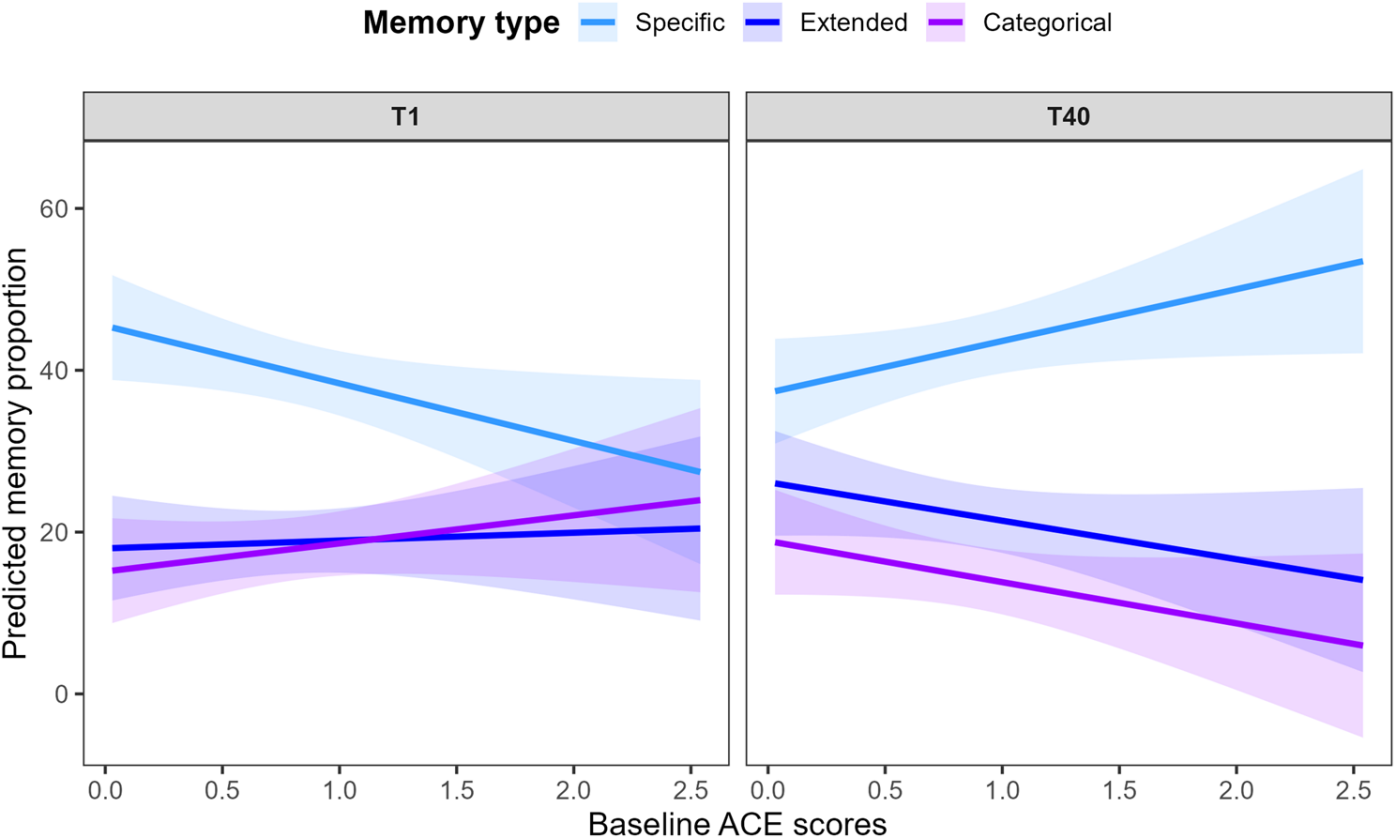
Interaction of time, memory type, and adverse childhood experiences (ACEs) in predicting autobiographical memory proportions. Predicted memory proportions (estimated marginal means) are shown for each memory type (specific, extended, categorical) across ACE scores, separately for the two time points (T1 and T40). The shaded areas represent 95% confidence intervals. The visualization illustrates how the association between ACEs and memory retrieval differs by memory type and time point. As indicated by the statistical simple slope analyses, the ACE-specific memory association shifts from negative at T1 to positive at T40, whereas no corresponding changes were observed for extended or categorical memories

The linear regression with time (T1 vs. T40), memory type (specific, categorical, extended), and ACEs as fixed effects explained a substantial amount of variance in memory proportions (adjusted *R²* = 0.366, *F*(11, 318) = 18.28, *p* < .001). The results are displayed in Table 3. To clarify the significant three-way interaction (*time × memory type × baseline ACEs*), we conducted follow-up analyses in two steps: (1) Estimated marginal means were compared to examine main and interaction effects of *time* and *memory type* (controlling for baseline ACEs). (2) Simple slope analyses were then performed to explore how ACEs predicted changes in each memory type across time. The resulting interaction pattern is illustrated in Fig. 2, which depicts model-based predicted memory proportions as a function of baseline ACE scores for each memory type and time point.

**Table 3:** Linear model predicting memory specificity

Predictor	B	SE	t	*p*
(Intercept)	45.487	3.368	13.506	< 0.001
time (T40)	-8.271	4.763	-1.736	0.083
categorical vs. specific	-30.359	4.763	-6.374	< 0.001
extended vs. specific	-27.498	4.763	-5.773	< 0.001
ACE_pre	-7.112	3.219	-2.210	0.028
time × categorical	12.037	6.736	1.787	0.075
time × extended	16.447	6.736	2.442	0.015
time × ACE_pre	13.513	4.552	2.969	0.003
categorical × ACE_pre	10.584	4.552	2.325	0.021
extended × ACE_pre	8.077	4.552	1.774	0.077
time × categorical × ACE_pre	-22.073	6.437	-3.429	< 0.001
time × extended × ACE_pre	-19.239	6.437	-2.989	0.003

Reference category: specific

#### Estimated marginal means contrasts

At the mean ACE level, estimated marginal means indicated no significant changes for specific memories from T1 to T40 (Δ = +3.22, *p* = .25), and no significant changes for extended (Δ = +3.31, *p* = .23) or categorical memories (Δ = − 3.51, *p* = .21). Follow-up difference-in-differences contrasts showed no significant differences in change across memory types (specific vs. extended or categorical; all *ps* > 0.23). However, pairwise contrasts revealed consistent differences between memory types at both time points: at T1, specific memories were recalled more frequently than extended (Δ = 20.63, *p* < .001) or categorical (Δ = 21.36, *p* < .001) memories. At T40, these differences remained significant (Δ specific – extended = 20.54, *p* < .001; Δ specific – categorical = 28.09, *p* < .001). These results confirm robust distinctions between memory types across therapy, with no overall time effect independent of ACEs.

#### Simple slope analyses (ACE × time × memory type)

For specific memories, higher ACE scores were associated with fewer memories at baseline (*B* = − 7.11, *p* = .028) but with more specific memories at follow-up (*B* = + 6.40, *p* = .048). The difference in ACE slopes between T1 and T40 was significant (ΔSlope = + 13.51, *p* = .003), indicating a reversal in the ACE-memory association over time, such that individuals with higher ACE scores showed a greater increase in AM specificity across the course of therapy.

For categorical memories, ACE slopes were positive but nonsignificant at T1 (*B* = + 3.47) and negative but nonsignificant at T40 (*B* = − 5.09). Nonetheless, ACE effects for categorical vs. specific memories differed significantly at T40 (*p* = .032), suggesting a divergence in how ACEs relate to these two memory types at follow-up.

For extended memories, ACEs showed no significant relationship at either time point (T1: *B* = + 0.97; T40: *B* = − 4.76). Yet at T40, the ACE slope for specific memories was significantly more positive than that for extended memories (*p* = .039).

#### Model extension with BDI change as covariate

To examine whether changes in depressive symptoms influenced AM proportions, we extended the initial regression model by including change in BDI scores as an additional covariate. Change scores for depressive symptoms were computed as the difference between post- and pre-treatment BDI scores (BDI post-treatment – BDI pre-treatment). This extension did not substantially alter the pattern of results: the model remained largely stable, and BDI change was not significantly associated with memory proportions (*B* = 0.05, *p* = .559). These findings indicate that the changes in memory specificity – especially those related to ACEs – appear to occur independently of improvements in depressive symptoms.

#### Model extension with ACE change scores

To explore whether changes in reported ACE scores were related to AM proportions, we ran an additional model in which baseline ACE scores were replaced with ACE change scores (ACE post – ACE pre). Replacing baseline ACE scores with ACE change scores did not substantially alter the overall pattern of results. ACE change was not significantly associated with memory proportions (*B* = − 4.05, *p* = .502), although a significant three-way interaction emerged for extended memories (time × memory type × ACE change: *B* = 29.68, *p* = .014).

#### Additional control analysis including sequence count

To account for variation in narrative output, we conducted a control analysis by including the total number of analyzed sequences per participant and time point as a covariate in the regression model. This variable was not a significant predictor of memory proportions (*B* = 0.081, *p* = .245), and the overall pattern of results remained unchanged. These findings suggest that differences in narrative production did not confound the main effects of time, memory type, or ACEs.

#### Summary

Taken together, estimated marginal means contrasts showed stable mean levels of memory types across therapy, whereas simple slope analyses revealed a dynamic interaction with ACEs: patients with higher ACE scores recalled fewer specific memories at baseline but exhibited greater improvements in specificity over the course of treatment. Exploratory analyses indicated that these effects were largely robust when considering changes in depressive symptoms or reported ACE scores, and were not explained by differences in narrative output.

### Post hoc power analysis

To evaluate the statistical sensitivity of the design, a post hoc power analysis was conducted using G*Power 3.1.9.7 [71]. The data were structured in a long format, with each participant (*n* = 55) contributing one entry per combination of memory type (specific, categorical, extended) and time point (T1, T40), resulting in a total of 330 observations. Based on the non-adjusted coefficient of determination of the regression model (*R²* = 0.387), the corresponding effect size was calculated as *f²* = *R²* / (1 - *R²*) = 0.631. Using this effect size, with eleven predictors, an alpha level of 0.05, and a sample size of *n* = 330, the achieved power (1 – *β*) was determined to be 1.00, indicating excellent sensitivity to detect existing effects.

## Discussion

### Summary of findings

To our knowledge, this study is the first to analyze autobiographical memories (AMs) reported during psychodynamic psychotherapy sessions. Its key contribution lies in the development and successful implementation of a novel method for capturing changes in AM specificity over the course of therapy. Applying this method revealed a significant three-way interaction between time, memory type, and adverse childhood experiences (ACEs). Specifically, patients with higher ACE scores showed fewer specific memories at baseline but demonstrated a more pronounced increase in memory specificity throughout therapy. Together, these findings suggest that psychodynamic psychotherapy may contribute to improvements in AM specificity among individuals with greater childhood adversity. This pattern aligns with previous research demonstrating the therapeutic benefits for AM functioning [27, 39, 72].

### Evaluation of the developed method

Our attempt to analyze autobiographical narratives in response to therapist questions in the natural setting of videotaped psychodynamic psychotherapy sessions was the first of its kind. We sought to address the shortcomings of commonly used tests of AM specificity, such as the artificiality of the cue-word method, which does not reflect memory retrieval in everyday life [73].

The memory retrieval process in our study differs from that in standard tests in that patients did not force themselves to respond according to the test instructions (i.e., to think of something as specific as possible in response to a cue word). Instead, they responded to the therapist’s questions without paying attention to the specificity of their narrative. This is closer to the direct emergence of AMs in everyday life than to generative retrieval in an experimental setting [10]. This may have implications for AM specificity, as directly retrieved memories tend to be more specific [74]. In addition, there was no time criterion as in the AMT to disrupt patients’ thinking [75]. Taken together, our study shows that findings previously obtained only in experimental settings are valid for the actual spoken word of patients in psychotherapy sessions.

### Frequencies of different types of autobiographical memories

In contrast to our initial expectation, categorical and extended AMs, as subtypes of overgeneral autobiographical memory (OGM), did not decrease during therapy. Although a positive effect of psychotherapy on AM specificity has been reported previously in the literature [28, 30], other studies have not supported these findings [32]. Our results indicate that changes in AM specificity may be more nuanced and dependent on individual differences such as childhood adversity, rather than reflecting a uniform reduction in OGM patterns over time.

There were significantly more analyzed sequences of autobiographical narratives at T1 than at T40. Given the natural course of psychotherapy, it is plausible that as the relationship with the therapist improves over time, patients will speak more freely and therapists will need to ask fewer questions to encourage and stimulate autobiographical recall. In addition, the proportion of sequences representing memory responses (specific, extended, categorical, semantic) was higher at T1 than at T40. One explanation for this observation could be that in the later therapy sessions there was now a shared knowledge of the patients’ autobiographical experiences, which were thus no longer elaborated.

It is important to note that the observed time course of AM categories from T1 to T40 could, at least in part, be influenced by changes in conversational style over time, as there was a reduction in the number of sequences representing memory responses. To account for such variation, we calculated the proportion of each memory category relative to the total number of memory responses, which helps control for differences in overall verbal output. Nonetheless, the influence of shifting interactional dynamics cannot be fully excluded, and both cognitive and conversational factors should be considered when interpreting the findings.

### The role of adverse childhood experiences (ACEs)

Adverse and protective childhood experiences (ACEs / PCEs) were assessed twice in our study, at baseline and after treatment. Patients reported more ACEs and fewer PCEs after treatment compared to baseline. This likely reflects increased disclosure, greater recall, or reinterpretation of prior experiences as therapy progresses. Such changes are consistent with the notion that patients’ AM specificity improves during therapy [30, 31], potentially allowing them to recall childhood experiences that they had previously forgotten or repressed. An alternative explanation would be a therapy-induced reappraisal of previously attenuated memories that are now perceived as more unfavorable than before [76–78]. This therapeutic effect, while clinically meaningful, complicates the use of ACEs as a fixed baseline predictor and should be carefully considered in interpreting longitudinal findings. To address this potential confound, we conducted an exploratory analysis in which ACE scores were replaced by ACE change scores. This substitution did not result in any significant changes in the overall findings.

In addition, ACEs at baseline were predictive of improvement in memory specificity during treatment. Specifically, patients with a higher burden of ACEs recalled fewer specific memories at the beginning of treatment, but showed greater improvements in memory specificity over the course of therapy. These findings suggest that individuals with greater childhood adversity may benefit particularly from psychotherapy in terms of AM specificity, despite initially showing reduced specificity.

One possible explanation for this finding may be that ACEs are associated with a greater deterioration in AM specificity, which may imply a greater potential for improvement with therapy [13, 21, 22, 47, 79–81]. Additionally, patients who have experienced more ACEs may generally benefit more from psychotherapy than others [82, 83]. An alternative explanation for the greater improvement in memory specificity among individuals with high ACEs involves functional avoidance [8]. OGM may serve as a way to avoid distressing content, especially in those with trauma histories. Through therapy, such avoidance may decrease, allowing for more specific recall. Further research is needed to clarify the potential relationship between ACEs and changes in AM over the course of psychotherapy.

### Limitations

An important limitation of this work is the subjectivity of the measurement method, as a rating always depends to some extent on the rater performing it [84]. This is inherent in qualitative approaches such as this one [85]. Nevertheless, provisions were made to improve the reproducibility by developing a detailed guideline for the rating, which can be found as Additional file (see Additional file 1). Additionally, we conducted a second rating of memory types on 30 videos containing 637 memory sequences. Interrater reliability (IRR) was moderate to good depending on weighting, with very good agreement in binary coding (specific vs. non-specific). These findings further support the robustness of our coding procedure across time points. Nevertheless, we recommend implementing structured rater training in future studies to optimize coding consistency.

Another possible limitation of our methodology is that psychodynamic techniques often involve inquiring when something remains unclear [33]. This may have systematically trained patients to be more precise in their narratives, thus limiting the extent of overgeneralization. However, this technical issue may also provide a therapeutic mechanism for improving AM specificity over the course of treatment.

Because our study was designed to be naturalistic, we did not include a control group. This limits our ability to draw conclusions about causality regarding the treatment. Future replications should include a waitlist or active control group to better understand the treatment’s impact on outcome parameters, particularly AM specificity and OGM.

Some literature suggests that memory specificity and memory detail are related but distinct constructs [73]. While our coding focused on whether memories referred to specific events, we did not assess the richness of episodic versus semantic detail within each memory. This is a relevant limitation, as more detailed narratives may reduce the number of memories reported per session, potentially confounding our results. Although distinguishing between new memories and added details might be difficult in naturalistic therapy sessions, this distinction could be addressed in future research. Applying adapted versions of the Autobiographical Interview [86] may allow for a more precise analysis of episodic and semantic detail in therapeutic narratives, despite the increased methodological complexity.

Another potential limitation concerns variability in the number of autobiographical narrative sequences analyzed across sessions. Since fewer sequences were available at T40 than at T1, changes in memory type proportions could theoretically reflect overall reductions in narrative output rather than specific shifts in memory specificity. To mitigate this issue, we calculated memory proportions using the total number of memory-containing sequences – including semantic associates – as the denominator, allowing for a consistent reference across time points. Furthermore, we conducted an additional control analysis in which the total number of sequences per session was included as a covariate in the regression model. This variable was not a significant predictor of memory proportions (*B* = 0.081, *p* = .245), and the overall pattern of results remained unchanged.

A further constraint of this study is that it does not include a direct comparison between memory retrieval in psychotherapy and performance on a standardized task such as the AMT. While our focus was to examine AM as it naturally occurs within the therapeutic context, future research could incorporate parallel AMT assessments to directly compare memory characteristics across structured and naturalistic settings and to further clarify the added value of studying memory in relational contexts.

### Questions for future research

Future research could further explore the distinction between the *ability* to retrieve specific AMs and the *tendency* to do so. Although our study does not directly separate these components, the therapeutic setting likely engages both. Comparing memory retrieval across structured tasks (e.g., AMT) and naturalistic contexts like therapy may clarify whether reduced specificity reflects cognitive limitations, motivational factors, or their interaction. In contrast, another interesting avenue for further research would be to assess the extent to which the recall and sharing of memories of varying specificity can influence treatment progress. Further studies using our newly developed method with larger sample sizes, for example using machine learning tools that can analyze larger amounts of data than human raters [87], would be helpful to gain more insight into the bidirectional relationship between psychotherapy and AM specificity.

## Conclusions

This study introduces a novel, ecologically valid method for assessing autobiographical memory (AM) specificity within real-life psychotherapy sessions. During outpatient psychodynamic psychotherapy of major depression, AM specificity appears to improve in patients with a higher burden of adverse childhood experiences (ACEs). The observed ACE-related gains in specificity apply to patients’ highly individualized narratives within psychotherapy sessions and are not just an effect that can be measured in experimental designs. Future studies should investigate the possible mechanisms of these findings and their potential therapeutic use.

## Supplementary Information

Below is the link to the electronic supplementary material.

Supplementary Material 1

## Data Availability

The data that support the findings of this study were collected at the outpatient training clinic for psychodynamic psychotherapy at the Heidelberg University Hospital, Germany (https://www.klinikum.uni-heidelberg.de/zentrum-fuer-psychosoziale-medizin-zpm/hip/heidelberger-institut-fuer-psychotherapie-hip). Restrictions apply to the availability of these data, which are not publicly available and cannot be shared due to restrictions by the Ethics Committee of the Heidelberg University. Data can however be requested from the author J. Nowak (jonathan.nowak@med.uni-heidelberg.de) upon reasonable request and with permission of the Ethics Committee of the Heidelberg University. The analysis code of our data analysis is publicly available at: 10.11588/DATA/9EJP6D.

## Abbreviations

AM: Autobiographical memory
OGM: Overgeneral autobiographical memory
AMT: Autobiographical memory test
ACEs: Adverse childhood experiences
PCEs: Protective childhood experiences
T1: First therapy session
T40: Fortieth therapy session

## References

[1] Ferrari AJ, Santomauro DF, Aali A, Abate YH, Abbafati C, Abbastabar H, et al. Global incidence, prevalence, years lived with disability (YLDs), disability-adjusted life-years (DALYs), and healthy life expectancy (HALE) for 371 diseases and injuries in 204 countries and territories and 811 subnational locations, 1990–2021: a systematic analysis for the global burden of disease study 2021. Lancet. 2024;403(10440):2133–61. 38642570 10.1016/S0140-6736(24)00757-8 PMC11122111

[2] Simon GE, Moise N, Mohr DC. Management of depression in adults: A review. JAMA. 2024;332(2):141–52. 38856993 10.1001/jama.2024.5756

[3] Arnone D, Karmegam SR, Östlundh L, Alkhyeli F, Alhammadi L, Alhammadi S, et al. Risk of suicidal behavior in patients with major depression and bipolar disorder - A systematic review and meta-analysis of registry-based studies. Neurosci Biobehav Rev. 2024;159:105594. 38368970 10.1016/j.neubiorev.2024.105594

[4] Greenberg PE, Fournier AA, Sisitsky T, Simes M, Berman R, Koenigsberg SH, et al. The economic burden of adults with major depressive disorder in the united States (2010 and 2018). PharmacoEconomics. 2021;39(6):653–65. 33950419 10.1007/s40273-021-01019-4 PMC8097130

[5] Weiss-Cowie S, Verhaeghen P, Duarte A. An updated account of overgeneral autobiographical memory in depression. Neurosci Biobehav Rev. 2023;149:105157. 37030646 10.1016/j.neubiorev.2023.105157

[6] Williams JM, Broadbent K. Autobiographical memory in suicide attempters. J Abnorm Psychol. 1986;95(2):144–9. 3711438 10.1037//0021-843x.95.2.144

[7] Williams JM, Scott J. Autobiographical memory in depression. Psychol Med. 1988;18(3):689–95. 3186869 10.1017/s0033291700008370

[8] Williams JM, Barnhofer T, Crane C, Herman D, Raes F, Watkins E, et al. Autobiographical memory specificity and emotional disorder. Psychol Bull. 2007;133(1):122–48. 17201573 10.1037/0033-2909.133.1.122 PMC2834574

[9] Hallford DJ, Rusanov D, Yeow JJE, Barry TJ. Overgeneral and specific autobiographical memory predict the course of depression: an updated meta-analysis. Psychol Med. 2021;51(6):909–26. 33875023 10.1017/S0033291721001343

[10] Conway MA, Pleydell-Pearce CW. The construction of autobiographical memories in the self-memory system. Psychol Rev. 2000;107(2):261–88. 10789197 10.1037/0033-295x.107.2.261

[11] Barry TJ, Hallford DJ, Takano K. Autobiographical memory impairments as a transdiagnostic feature of mental illness: A meta-analytic review of investigations into autobiographical memory specificity and overgenerality among people with psychiatric diagnoses. Psychol Bull. 2021;147(10):1054–74. 34968086 10.1037/bul0000345

[12] McLeod HJ, Wood N, Brewin CR. Autobiographical memory deficits in schizophrenia. Cogn Emot. 2006;20(3–4):536–47. 26529221 10.1080/02699930500342472

[13] Ono M, Devilly GJ, Shum DH. A meta-analytic review of overgeneral memory: the role of trauma history, mood, and the presence of posttraumatic stress disorder. Psychol Trauma. 2016;8(2):157–64. 25961867 10.1037/tra0000027

[14] Callahan JL, Maxwell K, Janis BM. The role of overgeneral memories in PTSD and implications for treatment. J Psychother Integr. 2019;29(1):32–41.

[15] Rosenbach C, Renneberg B. Remembering rejection: specificity and linguistic styles of autobiographical memories in borderline personality disorder and depression. J Behav Ther Exp Psychiatry. 2015;46:85–92. 25259768 10.1016/j.jbtep.2014.09.002

[16] Reinecke A, Rinck M, Becker ES, Hoyer J. Cognitive-behavior therapy resolves implicit fear associations in generalized anxiety disorder. Behav Res Ther. 2013;51(1):15–23. 23168327 10.1016/j.brat.2012.10.004

[17] Terhoeven V, Faschingbauer S, Huber J, Simon JJ, Herzog W, Friederich HC, et al. Autobiographical memory following weight gain in adult patients with anorexia nervosa: A longitudinal study. Eur Eat Disord Rev. 2024;32(4):809–23. 38558236 10.1002/erv.3091

[18] Terhoeven V, Nikendei C, Faschingbauer S, Huber J, Young KD, Bendszus M, et al. Neurophysiological correlates of disorder-related autobiographical memory in anorexia nervosa. Psychol Med. 2023;53(3):844–54. 34140047 10.1017/S003329172100221X

[19] Bomba M, Marfone M, Brivio E, Oggiano S, Broggi F, Neri F, et al. Autobiographical memory in adolescent girls with anorexia nervosa. Eur Eat Disorders Rev. 2014;22(6):479–86. 10.1002/erv.2321 25267565

[20] Sumner JA. The mechanisms underlying overgeneral autobiographical memory: an evaluative review of evidence for the CaR-FA-X model. Clin Psychol Rev. 2012;32(1):34–48. 22142837 10.1016/j.cpr.2011.10.003 PMC3246105

[21] Crane C, Heron J, Gunnell D, Lewis G, Evans J, Williams JM. Childhood traumatic events and adolescent overgeneral autobiographical memory: findings in a U.K. Cohort. J Behav Ther Exp Psychiatry. 2014;45(3):330–8. 24657714 10.1016/j.jbtep.2014.02.004 PMC4053588

[22] Hakamata Y, Mizukami S, Izawa S, Moriguchi Y, Hori H, Matsumoto N, et al. Childhood trauma affects autobiographical memory deficits through basal cortisol and prefrontal-extrastriate functional connectivity. Psychoneuroendocrinology. 2021;127:105172. 33831650 10.1016/j.psyneuen.2021.105172

[23] Fang J, Dong Y. Autobiographical memory disturbance in depression. Psychol Health Med. 2022;27(7):1618–26. 33870813 10.1080/13548506.2021.1916954

[24] Sumner JA, Griffith JW, Mineka S. Overgeneral autobiographical memory as a predictor of the course of depression: a meta-analysis. Behav Res Ther. 2010;48(7):614–25. 20399418 10.1016/j.brat.2010.03.013 PMC2878838

[25] Barry TJ, Hallford DJ, Rusanov D, Yeow JJE. Overgeneral and specific autobiographical memory predict the course of depression: an updated meta-analysis. Psychol Med. 2021;51(6):909–26. 33875023 10.1017/S0033291721001343

[26] Hallford DJ, Rusanov D, Yeow JJE, Barry TJ. Reduced specificity and increased overgenerality of autobiographical memory persist as cognitive vulnerabilities in remitted major depression: A meta-analysis. Clin Psychol Psychother. 2022;29(5):1515–29. 36129959 10.1002/cpp.2786 PMC9828164

[27] Hitchcock C, Smith AJ, Elliott R, O’Leary C, Gormley S, Parker J, et al. A randomized, controlled proof-of-concept trial evaluating durable effects of memory flexibility training (MemFlex) on autobiographical memory distortions and on relapse of recurrent major depressive disorder over 12 months. Behav Res Ther. 2021;140:103835. 33691266 10.1016/j.brat.2021.103835 PMC8047774

[28] Raes F, Williams JM, Hermans D. Reducing cognitive vulnerability to depression: a preliminary investigation of memory specificity training (MEST) in inpatients with depressive symptomatology. J Behav Ther Exp Psychiatry. 2009;40(1):24–38. 18407245 10.1016/j.jbtep.2008.03.001

[29] Barry TJ, Sze WY, Raes F. A meta-analysis and systematic review of memory specificity training (MeST) in the treatment of emotional disorders. Behav Res Ther. 2019;116:36–51. 30776658 10.1016/j.brat.2019.02.001

[30] McBride C, Segal Z, Kennedy S, Gemar M. Changes in autobiographical memory specificity following cognitive behavior therapy and pharmacotherapy for major depression. Psychopathology. 2007;40(3):147–52. 17318006 10.1159/000100003

[31] Williams JM, Teasdale JD, Segal ZV, Soulsby J. Mindfulness-based cognitive therapy reduces overgeneral autobiographical memory in formerly depressed patients. J Abnorm Psychol. 2000;109(1):150–5. 10740947 10.1037//0021-843x.109.1.150

[32] Hitchcock C, Rudokaite J, Haag C, Patel SD, Smith AJ, Kuhn I, et al. Autobiographical memory style and clinical outcomes following mindfulness-based cognitive therapy (MBCT): an individual patient data meta-analysis. Behav Res Ther. 2022;151:104048. 35121385 10.1016/j.brat.2022.104048 PMC7613018

[33] Leichsenring F, Abbass A, Heim N, Keefe JR, Kisely S, Luyten P, et al. The status of psychodynamic psychotherapy as an empirically supported treatment for common mental disorders - an umbrella review based on updated criteria. World Psychiatry. 2023;22(2):286–304. 37159376 10.1002/wps.21104 PMC10168167

[34] Summers RF, Barber JP. Psychodynamic therapy: a guide to evidence-based practice. New York ;: Guilford Press; 2010.

[35] Leichsenring F, Leibing E. Psychodynamic psychotherapy: a systematic review of techniques, indications and empirical evidence. Psychol Psychother. 2007;80(Pt 2):217–28. 17535596 10.1348/147608306X117394

[36] Debeer E, Hermans D, Raes F. Associations between components of rumination and autobiographical memory specificity as measured by a minimal instructions autobiographical memory test. Memory. 2009;17(8):892–903. 19882439 10.1080/09658210903376243

[37] Ridout N, Dritschel B, Matthews K, O’Carroll R. Autobiographical memory specificity in response to verbal and pictorial cues in clinical depression. J Behav Ther Exp Psychiatry. 2016;51:109–15. 26808234 10.1016/j.jbtep.2016.01.002

[38] Sumner JA, Mineka S, McAdams DP. Specificity in autobiographical memory narratives correlates with performance on the autobiographical memory test and prospectively predicts depressive symptoms. Memory. 2013;21(6):646–56. 23240988 10.1080/09658211.2012.746372 PMC3609943

[39] Forooshani S, Murray K, Izadikhah Z, Khawaja N. Identifying the most effective strategies for improving autobiographical memory specificity and its implications for mental health problems: a meta-analysis. Cogn Therapy Res. 2020;44.

[40] Boritz TZ, Angus L, Monette G, Hollis-Walker L. An empirical analysis of autobiographical memory specificity subtypes in brief emotion-focused and client-centered treatments of depression. Psychother Res. 2008;18(5):584–93. 18816008 10.1080/10503300802123245

[41] Boritz TZ, Angus L, Monette G, Hollis-Walker L, Warwar S. Narrative and emotion integration in psychotherapy: investigating the relationship between autobiographical memory specificity and expressed emotional arousal in brief emotion-focused and client-centred treatments of depression. Psychother Res. 2011;21(1):16–26. 20830647 10.1080/10503307.2010.504240

[42] Levitt H, Angus L. Psychotherapy process measure research and the evaluation of psychotherapy orientation: A narrative analysis. J Psychother Integr. 1999;9(3):279–300.

[43] Greenberg LS, Korman L. Assimilating emotion into psychotherapy integration. J Psychother Integr. 1993;3(3):249–65.

[44] Habermas T, Bluck S. Getting a life: the emergence of the life story in adolescence. Psychol Bull. 2000;126(5):748–69. 10989622 10.1037/0033-2909.126.5.748

[45] Felitti VJ, Anda RF, Nordenberg D, Williamson DF, Spitz AM, Edwards V, et al. Relationship of childhood abuse and household dysfunction to many of the leading causes of death in adults: the adverse childhood experiences (ACE) study. Am J Prev Med. 1998;14(4):245–58. 9635069 10.1016/s0749-3797(98)00017-8

[46] Kuyken W, Brewin CR. Autobiographical memory functioning in depression and reports of early abuse. J Abnorm Psychol. 1995;104(4):585–91. 8530760 10.1037//0021-843x.104.4.585

[47] Valentino K, Toth SL, Cicchetti D. Autobiographical memory functioning among abused, neglected, and nonmaltreated children: the overgeneral memory effect. J Child Psychol Psychiatry. 2009;50(8):1029–38. 19490313 10.1111/j.1469-7610.2009.02072.x PMC3513357

[48] Moore S, Zoellner L. Overgeneral autobiographical memory and traumatic events: an evaluative review. Psychol Bull. 2007;133:419–37. 17469985 10.1037/0033-2909.133.3.419 PMC2665927

[49] Weiste E, Peräkylä A. A comparative conversation analytic study of formulations in psychoanalysis and cognitive psychotherapy. Res Lang Social Interact. 2013;46:299–321.

[50] Mack C, Nikendei C, Ehrenthal JC, Spranz-Fogasy T. „[… Hab Ich glaub Ich die richtigen fragen gestellt. Therapeutische fragehandlungen in psychodiagnostischen Gesprächen. Volume 98. Mannheim: Institut für Deutsche Sprache; 2016. pp. S1–p.

[51] Spranz-Fogasy T, Graf E, Ehrenthal JC, Nikendei C. Requesting examples in psychodiagnostic interviews: therapists’ contribution to the sequential Co-construction of clients’ change. Communication Med. 2020;16:129–41. 10.1558/cam.34112 39916022

[52] Spranz-Fogasy T. Verstehensdokumentation in der medizinischen Kommunikation: Fragen und Antworten im Arzt-Patient-Gespräch. 2010. pp. 27–116.

[53] Vehviläinen S, Peräkylä A, Antaki C, Leudar I. A review of the conversational practices of psychotherapy. 2008.

[54] Gumz A, Horstkotte J, Kästner D. Das werkzeug des psychodynamischen Psychotherapeuten. Verbale interventionstypen Aus theoretischer und praxisorientierter perspektive. Zeitschrift für Psychosomatische Medizin Und Psychotherapie. 2014;60:219–37. 25331920 10.13109/zptm.2014.60.3.219

[55] Software VERBI. MAXQDA 2020. Berlin, Germany: VERBI Software; 2019.

[56] Gwet KL. Handbook of Inter-Rater reliability. 4 ed. Gaithersburg, MD: Advanced Analytics, LLC; 2014.

[57] Altman DG. Practical statistics for medical research. Boca Raton, FL: Chapman and Hall/CRC; 1990.

[58] Schauenburg H, Dinger U, Kriebel A, Huber J, Friederich H-C, Herzog W, et al. Zur Entwicklung Tiefenpsychologischer Ausbildungsinstitute Psychotherapeut. 2019;64(1):46–54.

[59] American Psychiatric Association. Diagnostic and Statistical Manual of Mental Disorders. 5th ed. Washington, DC2013.

[60] The Structured Clinical Interview for DSM-IV Axis I Disorders (SCID-I) and the Structured Clinical Interview for DSM-IV Axis II Disorders. SCID-II); 2004.

[61] Wittchen H-U, Zaudig M, Fydrich T. Strukturiertes klinisches Interview für DSM-IV: SKID; eine deutschsprachige, erweiterte Bearbeitung der amerikanischen Originalversion des SCID1997.

[62] Ehrenthal JC, Schauenburg H, Wagner FE, Dinger U, Volz M. [Development and evaluation of the questionnaire for the assessment of adverse and protective childhood experiences (APC)]. Psychiatr Prax. 2020;47(4):207–13. 32340049 10.1055/a-1123-1615

[63] Kühner C, Bürger C, Keller F, Hautzinger M. [Reliability and validity of the revised Beck depression inventory (BDI-II). Results from German samples]. Nervenarzt. 2007;78(6):651–6. 16832698 10.1007/s00115-006-2098-7

[64] Ehrenthal JC, Dinger U, Lamla A, Funken B, Schauenburg H. Evaluation der deutschsprachigen version des bindungsfragebogens „Experiences in close Relationships – Revised (ECR-RD). Psychother Psychosom Med Psychol. 2009;59(06):215–23. 18600614 10.1055/s-2008-1067425

[65] Thomas A, Brähler E, Strauss B. IIP-32: Entwicklung, validierung und normierung einer Kurzform des inventars Zur erfassung interpersonaler probleme. Diagnostica. 2011;57:68–83.

[66] Michael L, Burlingame G, Umphress V, Hansen N, Vermeersch D, Clouse G, et al. The reliability and validity of the outcome questionnaire. Clin Psychol Psychother. 1996;3:249–58.

[67] Löwe B, Spitzer R, Zipfel S, Herzog W. Autorisierte deutsche Version des Prime MD Patient Health Questionnaire (PHQ). Auflage New York: Pfizer ed2002.

[68] Bullinger M, Kirchberger I, Ware J. Der Deutsche SF-36 health survey Übersetzung und psychometrische Testung eines krankheitsübergreifenden instruments Zur erfassung der gesundheitsbezogenen Lebensqualität. J Public Health. 1995;3(1):21–36.

[69] Corp. I. IBM SPSS statistics for windows (Version 25.0). Armonk, NY, USA: IBM Corp; 2017.

[70] Team RC. R: A Language and environment for statistical computing (Version 4.4.2). Vienna, Austria: R Foundation for Statistical Computing; 2024.

[71] Faul F, Erdfelder E, Buchner A, Lang A-G. Statistical power analyses using G*Power 3.1: tests for correlation and regression analyses. Behav Res Methods. 2009;41(4):1149–60. 19897823 10.3758/BRM.41.4.1149

[72] Hitchcock C, Werner-Seidler A, Blackwell SE, Dalgleish T. Autobiographical episodic memory-based training for the treatment of mood, anxiety and stress-related disorders: A systematic review and meta-analysis. Clin Psychol Rev. 2017;52:92–107. 28086133 10.1016/j.cpr.2016.12.003

[73] Kopelman MD, Wilson BA, Baddeley AD. The autobiographical memory interview: a new assessment of autobiographical and personal semantic memory in amnesic patients. J Clin Exp Neuropsychol. 1989;11(5):724–44. 2808661 10.1080/01688638908400928

[74] Berntsen D. Voluntary and involuntary access to autobiographical memory. Memory. 1998;6(2):113–41. 9640425 10.1080/741942071

[75] Bartoli E, Smorti A. Facing the Language-Memory problem in the study of autobiographical memory. Integr Psychol Behav Sci. 2019;53(3):374–96. 29766475 10.1007/s12124-018-9434-x

[76] Ernst M, Beutel ME, Zwerenz R, Krakau L. Seeing the past in a new light: change in reports of childhood abuse and neglect before and after inpatient psychotherapy and its relevance for change in depression symptoms. Psychother Res. 2023;33(2):222–34. 35790188 10.1080/10503307.2022.2088313

[77] Schilling C, Weidner K, Schellong J, Joraschky P, Pöhlmann K. Patterns of childhood abuse and neglect as predictors of treatment outcome in inpatient psychotherapy: a typological approach. Psychopathology. 2015;48(2):91–100. 25501445 10.1159/000368121

[78] Kremers IP, Van Giezen AE, Van der Does AJW, Van Dyck R, Spinhoven P. Memory of childhood trauma before and after long-term psychological treatment of borderline personality disorder. J Behav Ther Exp Psychiatry. 2007;38(1):1–10. 16712781 10.1016/j.jbtep.2005.12.003

[79] Borrelli G, Lamberti Zanardi A, Scognamiglio C, Cinquegrana V, Perrella R. The relationship between childhood interpersonal and non-interpersonal trauma and autobiographical memory: a systematic review. Front Psychol. 2024;15:1328835. 38298520 10.3389/fpsyg.2024.1328835 PMC10827865

[80] Hitchcock C, Nixon RD, Weber N. A review of overgeneral memory in child psychopathology. Br J Clin Psychol. 2014;53(2):170–93. 24921070 10.1111/bjc.12034

[81] Goodman GS, Goldfarb D, Quas JA, Lyon A. Psychological counseling and accuracy of memory for child sexual abuse. Am Psychol. 2017;72(9):920–31. 29283641 10.1037/amp0000282

[82] Paivio SC. Stability of retrospective self-reports of child abuse and neglect before and after therapy for child abuse issues☆. Child Abuse Negl. 2001;25(8):1053–68. 11601597 10.1016/s0145-2134(01)00256-3

[83] Heinonen E, Knekt P, Härkänen T, Virtala E, Lindfors O. Childhood adversities as predictors of improvement in psychiatric symptoms and global functioning in solution-focused and short- and long-term psychodynamic psychotherapy during a 5-year follow-up. J Affect Disord. 2018;235:525–34. 29689505 10.1016/j.jad.2018.04.033

[84] Guastello SJ, Rieke ML, Guastello DD, Billings SW. Assessing the validity of computer-based test interpretations: rating reliability and individual differences among raters. J Pers Assess. 1992;58(1):79–89. 16370874 10.1207/s15327752jpa5801_7

[85] Austin Z, Sutton J. Qualitative research: getting started. Can J Hosp Pharm. 2014;67(6):436–40. 25548401 10.4212/cjhp.v67i6.1406 PMC4275140

[86] Levine B, Svoboda E, Hay JF, Winocur G, Moscovitch M. Aging and autobiographical memory: dissociating episodic from semantic retrieval. Psychol Aging. 2002;17(4):677–89. 12507363

[87] Takano K, Ueno M, Moriya J, Mori M, Nishiguchi Y, Raes F. Unraveling the linguistic nature of specific autobiographical memories using a computerized classification algorithm. Behav Res Methods. 2017;49(3):835–52. 27338931 10.3758/s13428-016-0753-x

