# Supplementary material for: Autobiographical narratives in major depression: changes in memory specificity during outpatient psychodynamic psychotherapy
Source: BMC Psychiatry. 2025 Dec 6;25:1144. doi: 10.1186/s12888-025-07666-7 (PMC12690838; doi:10.1186/s12888-025-07666-7)
Supplement: Supplementary file 1 — Supplementary Material 1 [file 12888_2025_7666_MOESM1_ESM.docx]

**Additional file 1**

**Guideline for Evaluating Autobiographical Narratives**

Sequences within a therapy session should be rated in chronological order to be able to identify repetitive information. If the same memory reappears later, it will not be rated twice. On the other hand, the content of previous sequences helps to provide context for the current sequence, which makes it easier to understand and rate the sequence.

Guiding principles:

- The assessment should be made from an outsider's point of view. The patient's feelings should be taken into account, but in relation to the disorder (e.g., something may seem very bad because of the patient's tendency to catastrophize, even though it is only a little bad).
- To quickly mention that something happened is not enough to count it as an autobiographical memory (AM). The patient must be mentally involved in the memory to count it.
- To evaluate the context of a memory, knowledge from earlier sequences of the therapy session can be taken into account.
- Even if we do not know the exact time of an AM, it can still be considered a specific AM, as long as it refers to a specific day.
- Remembering that something did not happen is not considered an AM.
- Reflecting on one's current mental state during an ongoing therapy session is not considered AM. Anything that happened before entering the therapy room counts as AM; anything that happened after entering the therapy room should not be counted.
- If these are not clearly stated, the rater may make assumptions about the time frame of the recall. For example, "The heating in the dormitory didn't work" may be interpreted as referring to the last few days/weeks and thus counted as an extended AM.
- Dreams count as AM.
- It is important to distinguish memories of the patient from memories of people known to the patient, as the latter should not be considered AM.

Repetitions and multiple AMs:

- Repetitions of an AM do not count as a new AM.
- If a sequence is counted as a repeat, but information is added that affects the evaluation of a previous sequence, that knowledge should be ignored because it was not reported within the 30-second time frame of the previous AM.
- If a specific AM is repeated, but another categorical or semantic AM is reported in the same sequence, the latter should be considered.
- If there are multiple descriptions within a 30-second sequence, the one with the highest specificity is counted as AM.
- If several independent specific AMs are mentioned within a 30-second sequence, the one with the most relevant content should be evaluated. If both appear to be covered at length, the first should be considered chronologically.

Semantic AMs:

- Opinions and attitudes can be rated as semantic AM, although the rating should be done rather strictly and only if it is actually a memory.
- Information about medications can also be classified as semantic AM, and in some situations even as specific AM.
- "It often happens to me that I start something and then don't want to do it anymore"

would be a classical semantic AM (and not categorical), since the speaker is not thinking of particular situations.

Therapist questions:

- If it is not clear whether it is a paraphrase or a question, one should consider whether the therapist's statement introduces a new aspect that can be explored further, or whether it is more an expression of understanding. In other words, whether the therapist would expect an answer beyond "yes.
- Generally, the tone of the therapist's voice is also taken into account, and sometimes decisions are made based on whether something is a paraphrase or an actual question. In difficult cases, this may depend on the context of the conversation.
- If the conversation is clearly not therapeutic but organizational, questions do not count (e.g., making the next appointment).
- Phrases like "I wonder if" are included because they are obviously meant as a question.

**Classification of therapist questions**

**#1 Open-ended question**

Definition: Questions that allow the patient to choose the topic. Often the first question in the therapy session, opening the conversation

Inclusion criteria:

- General invitation to speak
- Even if not explicitly stated as a question, especially at the beginning of the therapy session
- Also when a certain topic or feeling is suggested, but the possibilities for answering the question are still quite open-ended, e.g. "and you are not well"
- Also "How are you" if the question is meant to start the conversation
- An overlap with question types #2 and #4 is possible when the first question of a therapy session is related to a specific topic, but is still asked in an open-ended way

Exclusion criteria:

- No questions about the course of therapy if these questions are not open-ended, e.g. "How should I proceed now, should I ask a question?
- "How is your everyday life at the moment?" is a rather open-ended question, but still refers to a certain topic, in this case everyday life, and is therefore framed. Therefore, it is rated under #4.

Examples:

- “So, how are you doing?“
- "And on the other fronts?" in the sense of an open change of subject.

**#2 Focus in feelings**

Definition: Questions that address general or situational feelings. Past and present emotions may be of interest. The words “feeling” or “emotion” alone are not sufficient if they are not addressed.

Inclusion criteria:

- Both questions about general feelings and questions about situational feelings are included.
- The question may or may not refer to an AM.

Exclusion criteria:

- The focus should be on the emotional level, it is not enough to use a word from the lexical field of emotions if the question is aimed at something else
- No questions about someone else's feelings

Examples:

- “And how do you feel about being promoted to management?”
- "Are you worried?"
- "How come you said at the beginning of this session that you felt under pressure?”
- Also questions about energy levels: "But isn't it exhausting for you in such gatherings?"

**#3 Reflection on current AM**

Definition: This question type aims to explore the current AM in more detail to get the patient to reflect on it. The patient is most likely already thinking about an AM based on the previous conversation.

Inclusion criteria:

- Including hypothetical or problem-solving questions (often leads to the description of an AM when patients explain strategies they have used to solve things in the past)
- In general, questions that ask "why," e.g., "Why do you think your friend is against this?"

Exclusion criteria:

- No closed-ended questions that ask for facts (e.g., "How many pills do you take?", "How old is your son?")
- No closed-ended questions about whether the content was understood correctly (e.g., "Is this the situation you were referring to last time?”)
- No paraphrasing if it doesn't add a new aspect
- No closed-ended "either/or" questions
- No purely anamnestic interviews with closed-ended questions of medical interest (e.g., "What medications are you taking?")
- No questions about how to proceed in therapy (e.g., "Should I ask something now, or shall we move on?")
- No suggestions, no questions about the future
- Not purely organizational background questions such as "Is there a public library?" because they do not focus on the person's personal life

Examples:

- “What is new about it?“
- “What would you wish for?”
- "If you were to tell me what the two parts are called, what would you call them?"
- "Were the concerns you had about intimacy just blown away?"
- "Was that not understandable to you?”

**#4 Addressing new topic**

Definition: Question type that elicits an AM when the patient is unlikely to be thinking of an AM.

Inclusion criteria:

- The therapist addresses a particular situation, asking how something is or was
- It can be assumed that the patient was not yet mentally in the situation of interest, it is brought up by the therapist

Exclusion criteria:

- No questions about the present moment, e.g., "Where are you now, behind the wall, in front of the wall?”

Examples:

- “So, are there any aspects of yourself that you like?”
- "And what is A. like? Is she nice and friendly to everyone?"
- "What about dancing? Do you still do that? Is that important to you?"

**#5 Asking for specification**

Definition: The therapist asks for more specific information or examples about a previously superficially discussed topic.

Inclusion criteria:

- The patient mentions something but goes over it and the therapist focuses on it
- Phrases like "What do you mean by..." and "what does that mean..."
- A previously mentioned aspect is re-examined and further analyzed
- Questions about the most difficult, beautiful, important (etc.) situation or aspect

Exclusion criteria:

- Difference with #3: #5 simply says "please explain what you just said in more detail". #3, on the other hand, is more like "please explain this or that aspect to me in more detail," so the focus is a bit more directed.

Examples:

- “What do you mean by ‘little things at home’?“
- "I just wanted to ask again, in that letter you wrote, what kind of statement was it?"

**#6 Asking for generalization**

Definition: The therapist asks for a general statement or a similar experience derived from a specific situation. In terms of memory, a specific AM is linked to another by an associative criterion that can be of any specificity.

Examples:

- “You’ve done this before, haven’t you? Recently, a few months ago?“

Inclusion criteria:

- Extrapolation of the situation to other situations, e.g., "Would it be this bad in real life?
- Questions about transferability to other situations
- The patient's behavior toward the therapist is placed in an everyday context, e.g., "Do you feel the same way about others?"
- Something being discussed was there at another time, or reminds the patient of another time
- The patient is asked to step out of the situation and describe the problem in general terms, detached from the situation
- Questions about other moments on the timeline "Was it always like this?
- "I wonder what this means for us here in therapy" - shifting the level from the current conversation to the therapy as a whole
- Search for situations in which a discussed phenomenon has occurred

Exclusion criteria:

- Not if the question is asked about a specific situation to which the statement also applies, e.g., "Has anyone ever told you that you're volatile?” Then #4 is coded.
- Not when it comes to reflecting on the AM in the sense of "would you do it again" or finding a general explanation for the AM. Both are coded as #3.

Examples:

- "Did they get along with you before?"
- "You say that as if there were an area where he wasn't so generous?"
- "I'm still trying to figure out what that means, what does that mean for us here?"
- "Do you know anyone who is unstable? I'm still stuck on the idea that it would be difficult for you if someone thought you were volatile”
